# Air pollution particulate matter (PM2.5) prediction in South African cities using machine learning techniques

**DOI:** 10.3389/frai.2023.1230087

**Published:** 2023-10-10

**Authors:** Tshepang Duncan Morapedi, Ibidun Christiana Obagbuwa

**Affiliations:** Department of Computer Science and Information Technology, School of Natural and Applied Sciences, Sol Plaatje University, Kimberley, South Africa

**Keywords:** air pollution, pollutants, Particulate Matter (PM2.5), air quality, machine learning, data analysis, health

## Abstract

**Background:**

Air pollution contributes to the most severe environmental and health problems due to industrial emissions and atmosphere contamination, produced by climate and traffic factors, fossil fuel combustion, and industrial characteristics. Because this is a global issue, several nations have established control of air pollution stations in various cities to monitor pollutants like Nitrogen Dioxide (NO2), Ozone (O3), Sulfur Dioxide (SO2), Carbon Monoxide (CO), Particulate Matter (PM2.5, PM10), to notify inhabitants when pollution levels surpass the quality threshold. With the rise in air pollution, it is necessary to construct models to capture data on air pollutant concentrations. Compared to other parts of the world, Africa has a scarcity of reliable air quality sensors for monitoring and predicting Particulate Matter (PM2.5). This demonstrates the possibility of extending research in air pollution control.

**Methods:**

Machine learning techniques were utilized in this study to identify air pollution in terms of time, cost, and efficiency so that different scenarios and systems may select the optimal way for their needs. To assess and forecast the behavior of Particulate Matter (PM2.5), this study presented a Machine Learning approach that includes Cat Boost Regressor, Extreme Gradient Boosting Regressor, Random Forest Classifier, Logistic Regression, Support Vector Machine, K-Nearest Neighbor, and Decision Tree.

**Results:**

Cat Boost Regressor and Extreme Gradient Boosting Regressor were implemented to predict the latest PM2.5 concentrations for South African Cities with recording stations using past dated recordings, then the best performing model between the two is used to predict PM2.5 concentrations for South African Cities with no recording stations and also to predict future PM2.5 concentrations for South African Cities. K-Nearest Neighbor, Logistic Regression, Support Vector Machine, Decision Tree, and Random Forest Classifier were implemented to create a system predicting the Air Quality Index (AQI) Status.

**Conclusion:**

This study investigated various machine learning techniques for air pollution to analyze and predict air pollution behavior regarding air quality and air pollutants, detecting which areas are most affected in South African cities.

## 1. Introduction

In recent years, the industry's fast growth has been accompanied by air pollution, which kills millions of people yearly and gets widespread attention (Guo et al., 2020). According to the World Health Organization (WHO), about 90% of people breathe air that is contaminated and violates WHO air quality criteria (Bekkar et al., 2021; World Health Organization, 2021). Air pollution is a worldwide health issue, causing respiratory disorders, lung problems, eye problems, and skin diseases in people and affecting the ability of plants and animals to thrive. As a result, air pollution control and prevention have become major concerns. Factories' smoke exhaust, pollution caused by vehicles' exhaust, and power plants are the primary causes of air quality degradation (Sultana, 2019). (PM2.5, PM10), O3, SO2, CO, and NO2 are the five categories of air pollutants (Mao et al., 2021). PM2.5 is the most concerning air pollution component because these particles are small and light. They can stay in the atmosphere longer and easily bypass the filters in the human nose and throat (Akiladevi et al., 2020). PM2.5 is a standard air quality metric. However, it is usually measured with ground-based sensors (Jonathan et al., 2020). Many researchers focus on air pollution because of its increasing attention, and numerous important research papers are on it. Due to population and economic expansion, global energy consumption is steadily growing (Heydari et al., 2021).

Traditional statistical approaches have been frequently applied to solve air quality forecasting difficulties. These strategies are based on the principle of using historical data for learning; however, owing to the time-series data complexity and variance, they can produce poor estimates of air pollution. Several machine-learning algorithms have been developed during the last 60 years to aid in the resolution of complexity concerns (Ameer et al., 2019). Ensemble learning, MLR, SVM, RF, ANN, and other hybrid models are the primary machine learning approaches to combat air pollution (Bekkar et al., 2021). However, because the model selection is the focus of most prediction approaches and reasons for the change in air pollution concentrations are not analyzed by most present air quality prediction machine learning methods (Ameer et al., 2019). Furthermore, since contemporary deep learning frameworks are relatively adaptable, the model may need to be deep and sophisticated to match the Dataset. As a result, many weights in a deep neural network model may cause overfitting difficulties.

To assess and forecast the behavior of Particulate Matter (PM2.5), this study presents a Machine Learning approach that includes Cat Boost Regressor, Extreme Gradient Boosting Regressor, Random Forest Classifier, Logistic Regression, Support Vector Machine, K-Nearest Neighbor, and Decision Tree. This study summarizes the procedure of these methods to estimate the best solution for the corresponding requirement in any circumstance, to forecast air quality to raise public awareness about air quality degradation and its health effects.

The rest of the paper proceeds as follows: Section 2 presents the literature review, Section 3 presents the methodology used for the study, Section 4 presents the experiment and results, Section 5 shows the discussion of results, and Section 6 compares this work with existing research. Finally, Section 7 concludes the paper with a summary of the main points, future directions, and the study's limitations.

## 2. Literature review

According to Liao et al. (2020), no studies with complete adequate long-time intervals that include pollutant measurements from all sources, CTM (Chemistry-Transport Models), data assimilation products, driving meteorological fields, and emission sources. As a result, to progress, it will be required first to create such extensive benchmark datasets for testing learning algorithms and designing deep network topologies. They examined studies on methods such as RNN, LSTM, GRU, CNN (Convolutional Neural Network), SAE (Sparse Autoencoder), and DBN (Deep Belief Network) for Air Quality Forecasts in this paper. Finally, they determined that dealing with meteorological factors and pollution measurements from ground-level monitoring networks limits deep-learning research for air quality forecasts. They looked at attempts to use deep learning techniques to overcome the limitations of standard air quality forecasting methods that use chemistry-transport models (CTMs) or shallow statistical methods.

Ameer et al. (2019) studied and compared four current methods for predicting air pollution in smart cities in Machine Learning Techniques for Predicting Air Quality comparative analysis. The methods were RF regression, GBR, DT (Decision Tree) regression, MLP (Multi-Layer Perceptron) regression, and RF regression emerged as the best. They identified which of the compared techniques used to predict Air Pollution is the best. They did not discuss data handling. Sultana compared air pollution detecting techniques using image processing, machine learning, and deep learning approaches, where they evaluated these three methods used to detect air pollution and better compare estimates, how they operate, and are processed in the air pollution detection (Sultana, 2019). Finally, they determined that the deep learning technique outperforms the other two regarding efficacy and accuracy. However, it necessitates a large dataset, and as the accuracy level rises, so does the total expenditure and cost. They considered three procedures (Image Processing, Machine Learning, and Deep Learning) used to detect air pollution and estimate a better comparison of how they work and are processed in air pollution detection. Data implementation was not discussed (Sultana, 2019).

Guo et al. developed an EN model to forecast PM2.5 concentrations based on previous PM2.5 concentrations, meteorological data, and time stamp data. RNN, GRU, LSTM, and NN (Neural Network) were among the optimum algorithms employed. Human activities and topographical data were missing from the study (Guo et al., 2020). The findings showed that the suggested technique beats existing algorithms in terms of performance. Mao et al. used graph convolution and LSTM networks to create and present a spatiotemporal modeling hybrid deep learning framework to forecast various air contaminants (Mao et al., 2021). Models such as MLR and LSTM networks were employed. The findings revealed that the distribution of errors in space, to some extent, corresponds to the spatiotemporal correlation strength distribution, highlighting the necessity of spatiotemporal dependency modeling for pollutant prediction. They did not discuss data implementation. Heydari et al. (2021) anticipated and assessed air pollution from Combined Cycle Power Plants by creating a novel hybrid intelligence model based on MVO (Multi-Verse Optimizer) algorithm and LSTM. They applied the method only to observe the correlation coefficient of NO2 and SO2 pollutants.

Xayasouk and Lee proposed a deep-learning-based technique for fine dust prediction. They utilized the deep-learning algorithm to construct a spatiotemporal prediction framework that considers the Dataset's temporal and geographical relationships during the modeling process (Xayasouk and Lee, 2018). To train and evaluate the data, they employed the Stacked Encoders model, which is unsuitable for learning and training the time series data (Xayasouk and Lee, 2018). Abdellatif et al. created a CNN-LSTM that can be utilized to estimate air quality and can efficiently conduct Spatiotemporal prediction (Bekkar et al., 2021). Deep learning models such as LSTM, CNN, GRU, CNN-GRU, CNN-LSTM, Bi-LSTM, and RNN were utilized (Bekkar et al., 2021). The model can efficiently extract data from temporal and spatial aspects using CNN and LSTM, and it also has excellent accuracy and stability, according to the findings of this work. They did not discuss the processing time. Aarthi et al. (2020) stated that Environmentalists and the government aided in framing air quality standards and regulations based on hazardous and pathogenic air exposure and health-related risks to human welfare. The processed datasets were used to generate a function that plots the training and validation data for several models, including SV (Support Vector), Lasso, Linear, and DT regression. The authors found that their project raised public awareness, assisted environmentalists and the government in creating air quality standards and regulations based on hazardous and pathogenic air exposure and health-related dangers to human welfare, and discussed the health effects of air quality degradation. They used a decision tree in this experiment, which is not a suitable classifier for time series data (Aarthi et al., 2020).

Aditya et al. (2018) suggested an approach that would assist ordinary people and meteorologists in detecting and forecasting pollution levels and responding appropriately. Logistic Regression and Autoregression were employed as machine-learning regression approaches. This will also assist individuals in establishing a data source for small towns, which are sometimes overlooked compared to major cities. Logistic Regression performed well on a prediction but failed to explain the constraints (Aditya et al., 2018). Balasubramanian et al. (2021) developed a technique to anticipate the following 5 h' Air Quality Index. They employed a Linear regression model, an SV regression Model, and RF regression Model for data analysis. According to the researchers, Machine Learning algorithms were used to anticipate the AQI (Air Quality Index) values for the following 5 h (Balasubramanian et al., 2021). The Stacking Ensemble model has the lowest RSME (Root Mean Squared Error) value when all the models' RMSE (Root Mean Square Error) values are compared. As a result, this model was picked to anticipate the following 5 h' Air Quality Index. They did not thoroughly discuss data handling. Dobrea et al. developed a technique that calculates the number of atmospheric pollutants (PM2.5 and PM10) (Dobrea et al., 2020). Support Vector Regression, Autoregression Integrated Moving Average, and LSTM are the models employed. After a comparison of data analysis methods and Machine Learning algorithms for estimating atmospheric pollutants (PM10 and PM2.5), it was determined that the Support Vector Regression and ARIMA (Auto Regressive Integrated Moving Average) algorithms are the most suitable for forecasting air pollutants concentrations, with correlation coefficients of 96.6% and 92.1% for PM10 and PM2.5, respectively (Dobrea et al., 2020). The experiment only focused on one factor of air pollution.

Akiladevi et al. (2020) proposed a technique for developing an air quality forecasting system that can anticipate main contaminants in various locations. To assess the Dataset's performance, ML (Machine Learning) methods such as LR (Linear Regression), NB (Naïve Bayes), SVM, RF, KNN (K-Nearest Neighbor), and DT were utilized. Performance measurement factors such as accuracy, recall, f1-score, Specificity, and Sensitivity were computed for each method. For each technique, confusion matrix parameters such as TP (True Positive), TN (True Negative), FP (False Positive), and FN (False Negative) were determined. LR had a 98% accuracy, NB had a 95% accuracy, RF had a 99% accuracy, SVM had a 70% accuracy, K-NN had a 97% accuracy, and DT had a 100% accuracy. Out of these six ML algorithms, the Decision Tree approach had the best accuracy (Akiladevi et al., 2020). The decision Tree was not a good time series data classifier, so it performed well in this research. Bui et al. (2018) proposed a deep learning technique for air quality index predictions. The Encoder-Decoder paradigm was employed, as well as Long Short-Term Memory units. Based on historical meteorological data, their suggested model produced substantial results in predicting PM2.5 AQI for the long term. The accuracy was discussed but not the processing time.

Taylan et al. (2021) mentioned that to minimize respiratory and cardiovascular deaths, researchers developed a method that is feasible, robust, and capable of evaluating pollutants' cumulative effect inside metropolitan areas. They employed the Non-linear Autoregressive with External (NARX) Input and the Levenberg–Marquardt (LM) Algorithm. They concluded that managing air pollution entails establishing capacity and monitoring ground-based networks and systems to make suitable strategic and operational decisions. Quality assurance and control, modeling methodologies, and institutional competencies are all required to implement these initiatives. The Dataset used was limited.

Kalajdjieski et al. (2020) developed a data fusion method for using multi-modal data such as weather and pollution measurements obtained by sensors and picture data collected by cameras. Basic Convolutional Neural Network, Residual Network Model, Inception Model, and Custom pre-trained Inception were among the predictive models tested. Their trials reveal that our bespoke pre-trained inception model, paired with their data preparation strategy, outperforms known state-of-the-art approaches in accuracy (Kalajdjieski et al., 2020). The model used was biased. Saleh et al. (2016) developed a model for predicting CO2 emissions from energy. The Support Vector Machine model was utilized. They concluded that a lower RMSE (Root Mean Square Error) value must be produced when the prediction model's accuracy is good. It can assist the management in developing policies or making decisions to limit the negative environmental impact throughout the manufacturing process by monitoring energy use. The experiment only focused on CO2 (Saleh et al., 2016).

Popa et al. developed a system model that forecasts temperature changes in a densely populated area of Bucharest, Romania. They employed LR, SVM with Gaussian kernel, and Gaussian process regression with the exponential kernel as well as other techniques (Popa et al., 2021). They concluded that future studies might combine the current findings with camera photos to assess and anticipate air pollution in various large cities or establish a platform to provide traffic suggestions based on air pollution predictions. They only used linear methods for classification.

Based on the reviewed literature on Machine Learning Applications in Air Pollution. To the best of our knowledge, no work was done involving the analysis and prediction of air pollution in South Africa. Many have been done in countries like China (Moursi et al., 2019; Guo et al., 2020; Harishkumar et al., 2020; Balasubramanian et al., 2021; Bekkar et al., 2021; World Health Organization, 2021), India (Aditya et al., 2018; Sultana, 2019; Aarthi et al., 2020; Akiladevi et al., 2020; Masood and Ahmad, 2020), Korea (Bui et al., 2018; Xayasouk and Lee, 2018; Yang et al., 2020), and Iran (Zamani Joharestani et al., 2019). The proposed method in this study will analyze and predict the behavior of PM2.5, monitor a period of historical levels and correlation analysis for future predictions of PM2.5 levels in cities of South Africa and evaluate the models used to find the best that will be used to measure the performance of the Dataset.

## 3. Methodology

This study used the Anaconda Navigator (Jupyter Notebook) and an AMD Ryzen 7 5700U computer with 8GB of RAM and a 1.80 GHz Radeon graphics processor. Python 3.6 exposed the proposed machine learning models to data cleaning and feature extraction for training and testing models. This study aims to investigate various machine learning approaches to air pollution, to analyse and predict air pollution behavior in terms of air quality and air pollutants (PM2.5), detecting which areas are most affected in South African cities. All the graphs in this chapter are created using Python. The data was handled using Pandas, and the charts were plotted with Matplotlib and Seaborn.

### 3.1. Air pollution methodology approach

This study aims to forecast the concentration of a particular substance (PM2.5) in South Africa. Most metropolitan people can suffer adverse effects from exposure to air pollutants like PM2.5 in ambient air. When pollutant concentrations exceed an air quality limit, we pay closer attention. Determining whether the PM2.5 concentration surpasses a specific threshold is the focus of the problem. There are several classification models in use. The proposed models used other air pollutants as initial features and meteorological data gathered at various heights above the ground. There are many features when the multiple periods of these features are considered. Therefore, we reduce the dimensionality of the data before using the classification models. The resampling technique is also used to manage an imbalanced data collection like ours. Next, a complete discussion of evaluation metrics follows.

### 3.2. Data understanding

The Dataset used is available at: https://aqicn.org/data-platform/covid19/. About the Dataset: The average (median) of numerous stations was used to compile the statistics for each main city. Each air pollution species' data set includes the minimum, maximum, median, standard deviation, and meteorological data. The US EPA (United State Environmental Protection Agency) standard is applied to all air pollutant species (i.e., no raw concentrations). All dates are in UTC (Coordinated Universal Time). The number of samples used to calculate the median and standard deviation is listed in the count column. PM2.5 is a unit of measurement for tiny inhalable particles having dimensions of 2.5 micrometers or less. High levels of PM2.5 have been linked to respiratory problems and other harmful health consequences, and they can constitute a serious health risk to residents. PM2.5 is a standard air quality metric; however, it is usually measured with ground-based sensors. This Dataset provides daily pollution estimates from January 2015 to February 2022 for 386 nations worldwide. The clusters in South African cities will be sampled from this Dataset. The Dataset includes (Middelburg, Pretoria, East London, Johannesburg, Bloemfontein, Cape Town, Vereeniging, Durban, Klerksdorp, Richards Bay, Port Elizabeth, and Worcester) which are considered stations for South Africa. The estimations will be derived using a model that has been trained using previous data from pollution sensor sites. Several global layers will be used as inputs to the model, including data from Sentinel 5P and meteorological details. The additional global layers are also obtained from the same Dataset whose link is provided above. To get the exact data for new locations, a dataset with a list of South African cities from https://simplemaps.com/data/za-cities is used, but the same process is repeated for other locations as well. The population centers are found using a custom Google Earth Engine script, available here: https://code.earthengine.google.com/6dc3cd0c9cf91ba69592c5ce4c54ff55.

Table 1 depict the attributes of the Dataset used for this work. Table 1 shows the attributes of the original Dataset, illustrates the attributes of the Dataset after sampling, and the attributes of the Dataset with a list of South African cities.

**Table 1:** Attributes of dataset.

**Attribute**	**Description**
**Attributes of the original dataset**	
Date	Contains the date of the recorded concentration
Country	Contains the country of the City of the recorded concentration
City	Contains the City of the recorded concentration
Specie	Contains the name of the of the pollutants (NO2, SO2, O3, CO, PM2.5, PM10)
Min	Contains the minimum concentration of the pollutant on the given date
Max	Contains the maximum concentration of the pollutant on the given date
Median	Contains the median of the concentration of the pollutant on the given date
Variance	Contains the variance of the concentration of the pollutant on the given date
**Attributes of the dataset after sampling**	
Date	Contains the date of the recorded concentration
City	Contains the South African City of the recorded concentration
Median_PM25	Contains the median concentration of the PM2.5
Lat	Contains the latitude of the City given
Long	Contains the longitude of the City given
**Attributes for a dataset with a list of South African cities**	
City	The name of the city/town
Lat	The latitude of the city/town
Lng	The longitude of the city/town
Country	The name of the city/town's country
Admin Name	The name of the highest-level administration region of the city town
Population	An estimate of the city's urban population
id	A 10-digit unique id generated by SimpleMaps

### 3.3. Research design

This research adopts the deductive approach adopted from the Positivism concept to use an experimental design to carry out cluster analysis for:

(i) Data Pre-processing: Missing values, Label Encoding, Normalization.

Data pre-processing was used to convert the raw data into an understandable format because the data in the real world is incomplete, noisy, and inconsistent. The generalized Dataset undergoes pre-processing, which helps recover missing, null, and duplicate values and convert the data into the numeric format.

Missing values are filled using the mean of the PM2.5 Median. Time Series Cross Validation is used to prevent overfitting and evaluate model performance.

**Figure 1:**
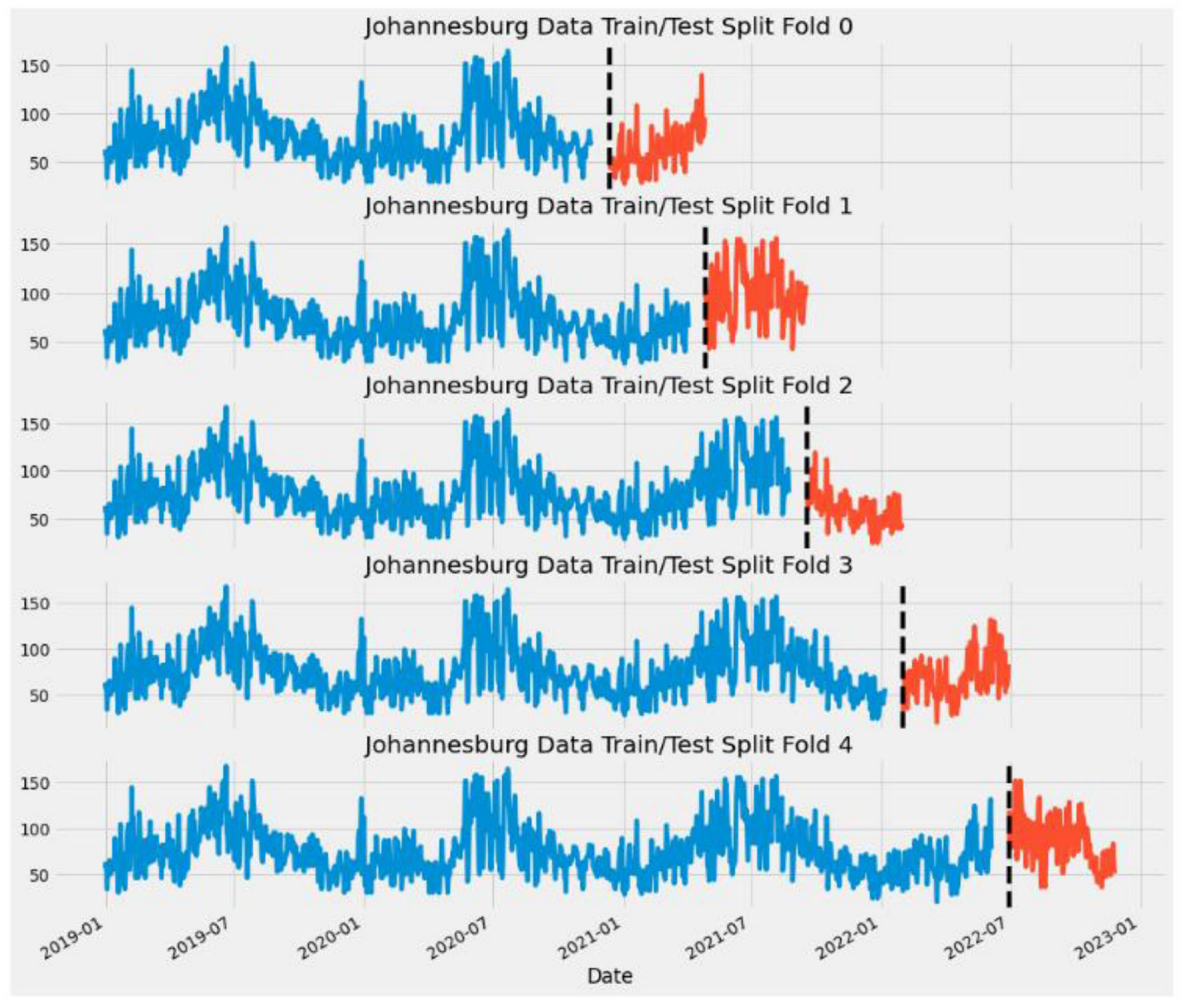
Applying time series cross validation.

Figure 1 shows one of the cities after applying the Time Series Cross Validation with 5-folds.

(ii) Feature Selection: Air Quality Feature, Meteorological Feature, and Correlation Analysis in a quantitative study, since there is an involvement of numerical data and experiments, and they are part of the quantitative research.

The PM2.5 concentrations of the South African Cities are sampled from the original Dataset, then merged with the Meteorological Data and the population centers found using the location coordinates.

(iii) Data Split: Train Set and Test Set.

The Dataset was split into training and testing datasets. Generally, by default, the Dataset is split in the ratio of 80:20, but in this system model, the Dataset is split by the date. The Train Set consists of the concentrations dated before' 01-01-2022′, and the Test Set consists of those dated on and after' 01-01-2022′.

**Figure 2:**
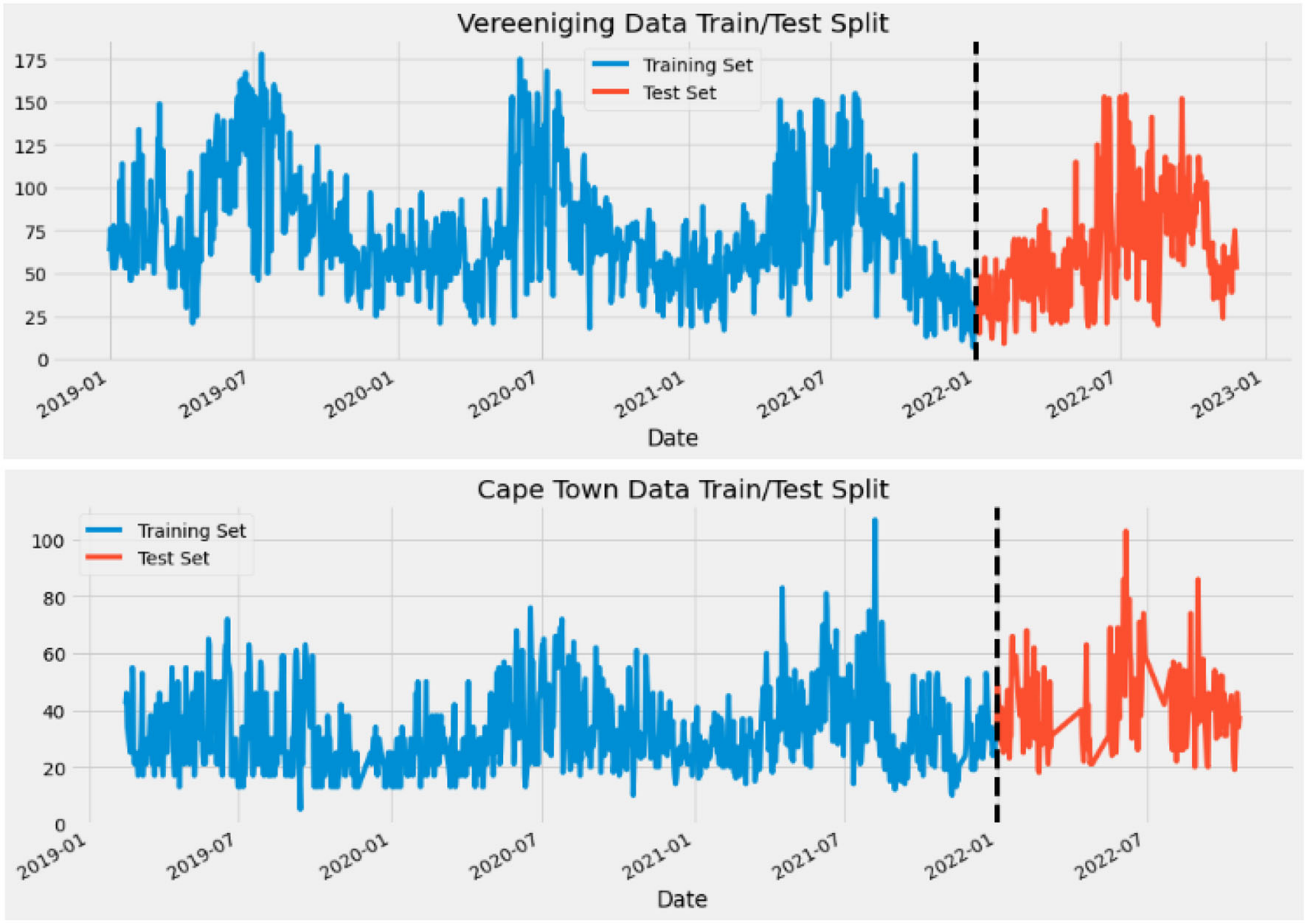
How data was split before making predictions.

Figure 2 shows the split data with 2 of the 12 cities.

(iv) Performance Evaluation

The Dataset is trained by applying ML algorithms such as Cat Boost Regressor, Extreme Gradient Boosting Regressor, K-Nearest Neighbor, Logistic Regression, Support Vector Machine, Decision Tree, and Random Forest Classifier.

The performance measurement parameters used in this work are as follows:

1. Precision:
  Precision is defined as the ratio of a true positive (TP) divided by the sum of a true positive (TP) and a false positive (FP).
  (1) $$\begin{array}{l} Precision=\frac{TP}{\left(TP+FP\right)} \end{array}$$
2. Recall:
  The recall is defined as the ratio of a true positive (TP) divided by the sum of a true positive (TP) and a false negative (FN).
  (2) $$\begin{array}{l} Recall=\frac{TP}{TP+FN} \end{array}$$
3. F1-score:
  F1 score is defined as the mean between precision and recall.
  (3) $$\begin{array}{l} F1=\frac{TP}{\begin{array}{c} TP+\frac{1}{2}\left(FP+FN\right) \end{array}} \end{array}$$
4. Specificity:
  Specificity is defined as the ratio of a true negative (TN) divided by the sum of a true negative (TN) and a false positive (FP).
  (4) $$\begin{array}{l} specificity=\frac{TN}{\left(TN+FP\right)} \end{array}$$
5. Sensitivity:
  Sensitivity is the true positive (TP) ratio divided by the sum of a true positive and false negative.
  (5) $$\begin{array}{l} sensitivity=\frac{TP}{\left(TP+FN\right)} \end{array}$$
6. Confusion matrix:
  A confusion matrix is represented as a table used to describe the performance of the classification model on a test dataset for which the correct values are known.

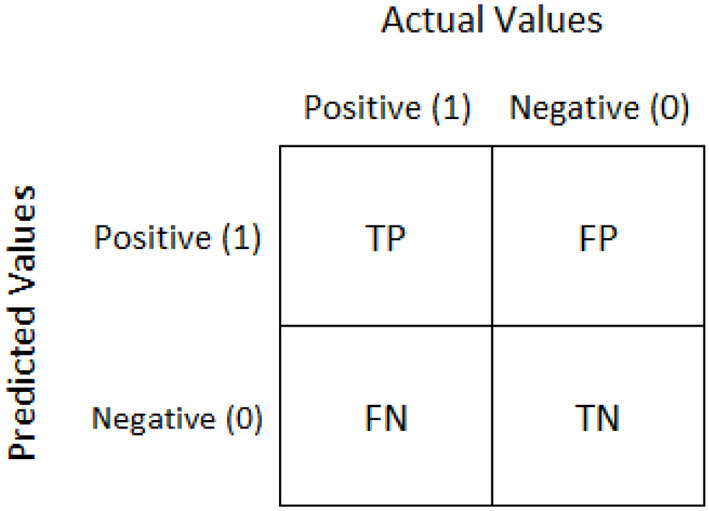

7. Mean Square Error
  The Mean Square Error (MSE) measures the error in statistical models using the average squared difference between actual and predicted values.
  (6) $$\begin{array}{l} MSE=\frac{1}{N}\overset{N}{\underset{i=1}{\sum }}{\left(y_{i}-ŷ\right)}^{2} \end{array}$$
8. Mean Absolute Error
  The Mean Absolute Error (MAE) measures the average magnitude of the errors between the actual and predicted values.
  (7) $$\begin{array}{l} MAE=\frac{1}{N}\overset{N}{\underset{i=1}{\sum }}|y_{i}-ŷ| \end{array}$$
9. Root Mean Square Error
  The Root Mean Square Error (RMSE) measures the average difference between a statistical model's predicted and actual values.
  (8) $$\begin{array}{l} RMSE=\sqrt{MSE}=\sqrt{\frac{1}{N}\overset{N}{\underset{i=1}{\sum }}(y_{i}-ŷ{)}^{2}} \end{array}$$

(v) Training and Testing the Model

Cross-validation trained and tested the XGB model with five splits, a test size of 150, and a gap of 24. With features being the day of the year, and days of the week, with lag variables and the target being the median of PM2.5. The regressor base score was set to 0.5, with booster as the gradient boosting tree, with 1,000 estimates, three max depths, and a learning rate of 0.01.


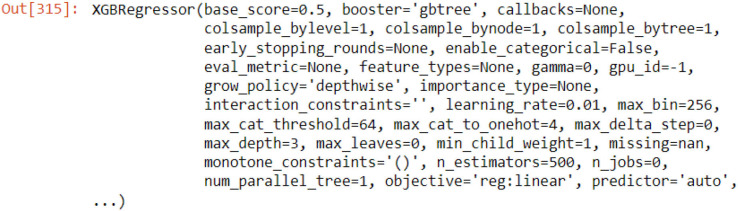

(vi) Predictions.

1. Predicting the latest PM2.5 concentrations for South African Cities with recording stations using past-dated recordings.
2. Predicting PM2.5 concentrations for South African Cities with no recording stations.
3. Predicting Future PM2.5 Concentrations for South African Cities.
4. Predicting the Air Quality Index (AQI) Status.

### 3.4. Data transformation

The clusters in South African cities were sampled from the original Dataset. The clustered Dataset includes cities like (Middelburg, Pretoria, East London, Johannesburg, Bloemfontein, Cape Town, Vereeniging, Durban, Klerksdorp, Richards Bay, Port Elizabeth, and Worcester) which are considered stations for South Africa.

**Figure 3:**
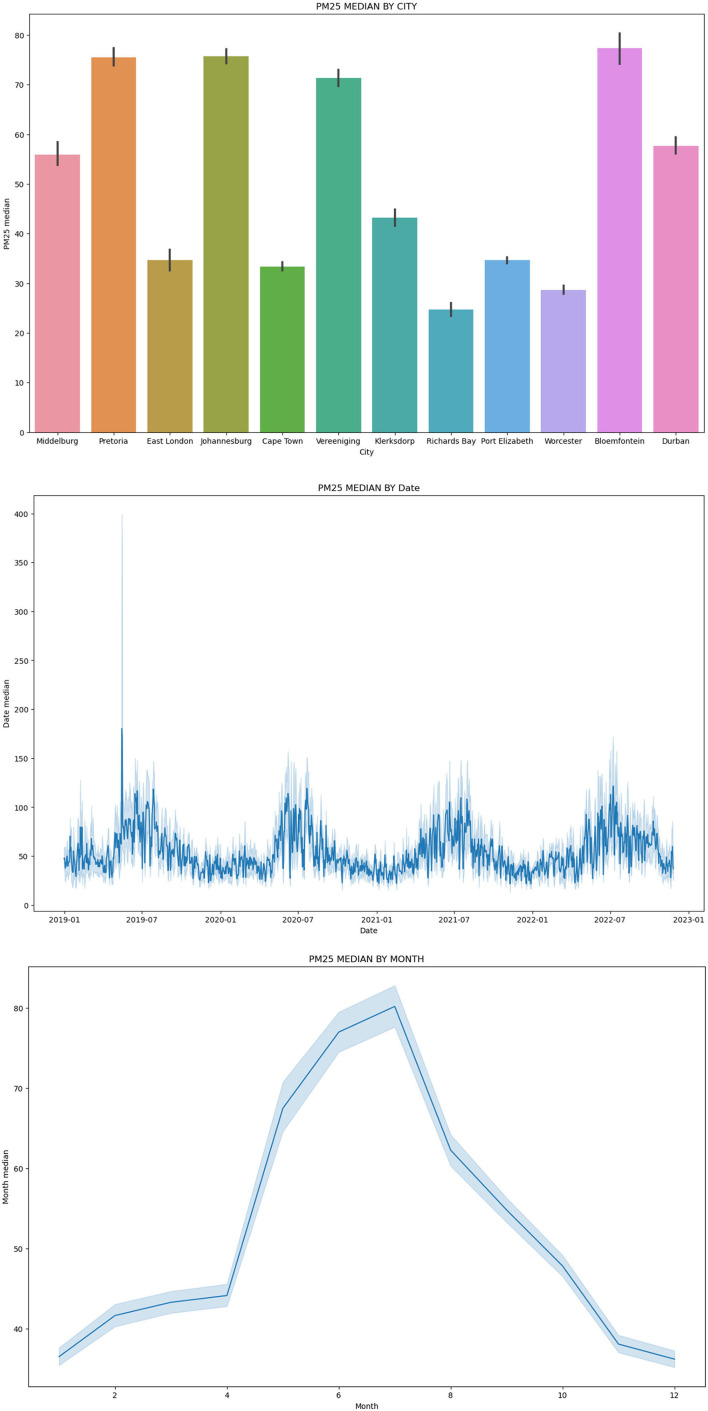
Clustered data by city, date, and month.

Figure 3 shows how the clustered data looks by City, Date, and Month.

**Figure 4:**
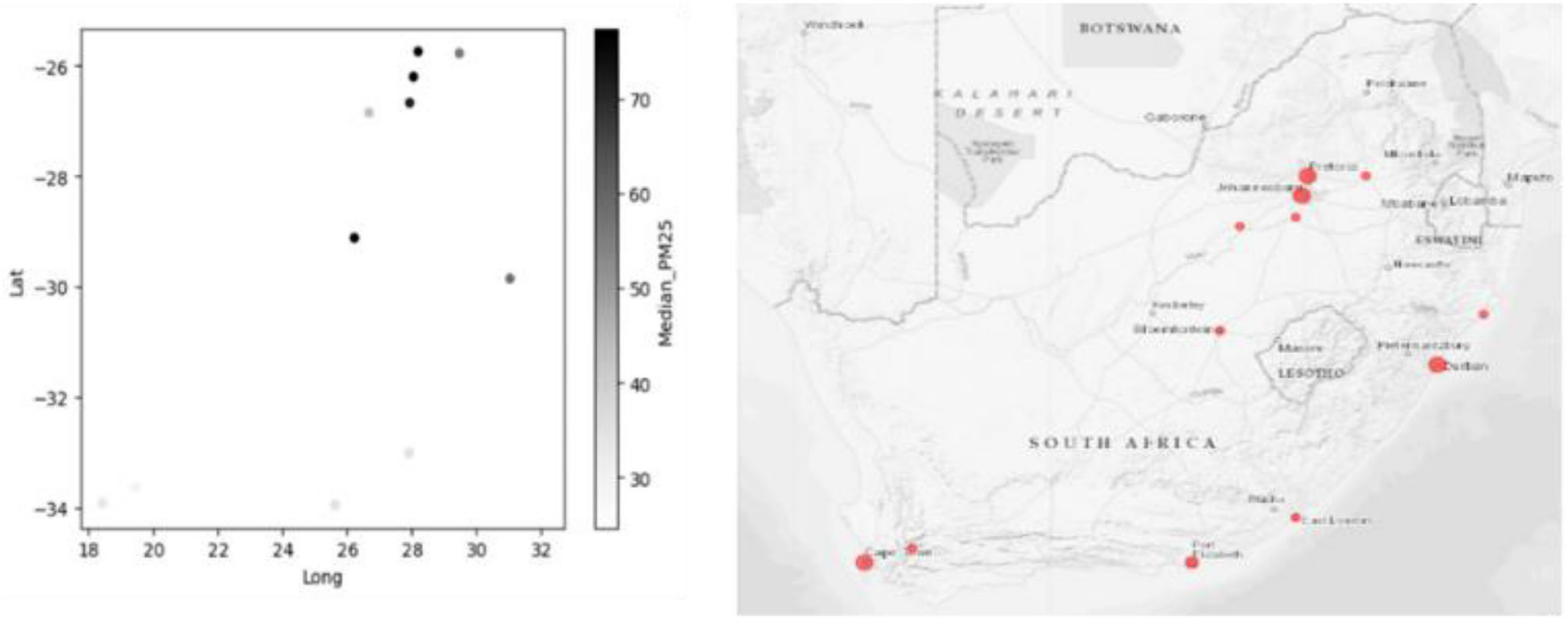
South African cities locations based on the maps.

From the clustered Dataset, only the data of PM2.5 was selected and used for predictions. Figure 4, on the right, is the original map of South Africa, with the cities included in the Dataset plotted. On the left is the map plot according to the Median_PM25 concentrations, plotted based on the Longitude and Latitude of the South African Cities.

The saved data with air quality measurements were augmented with satellite data via GEE (Google Earth Engine), getting it into a state that is ready for modeling to get the exact data for a new location which is essential when making predictions with no stations (ones which were not included in the Dataset.

### 3.5. Modeling

**Figure 5:**
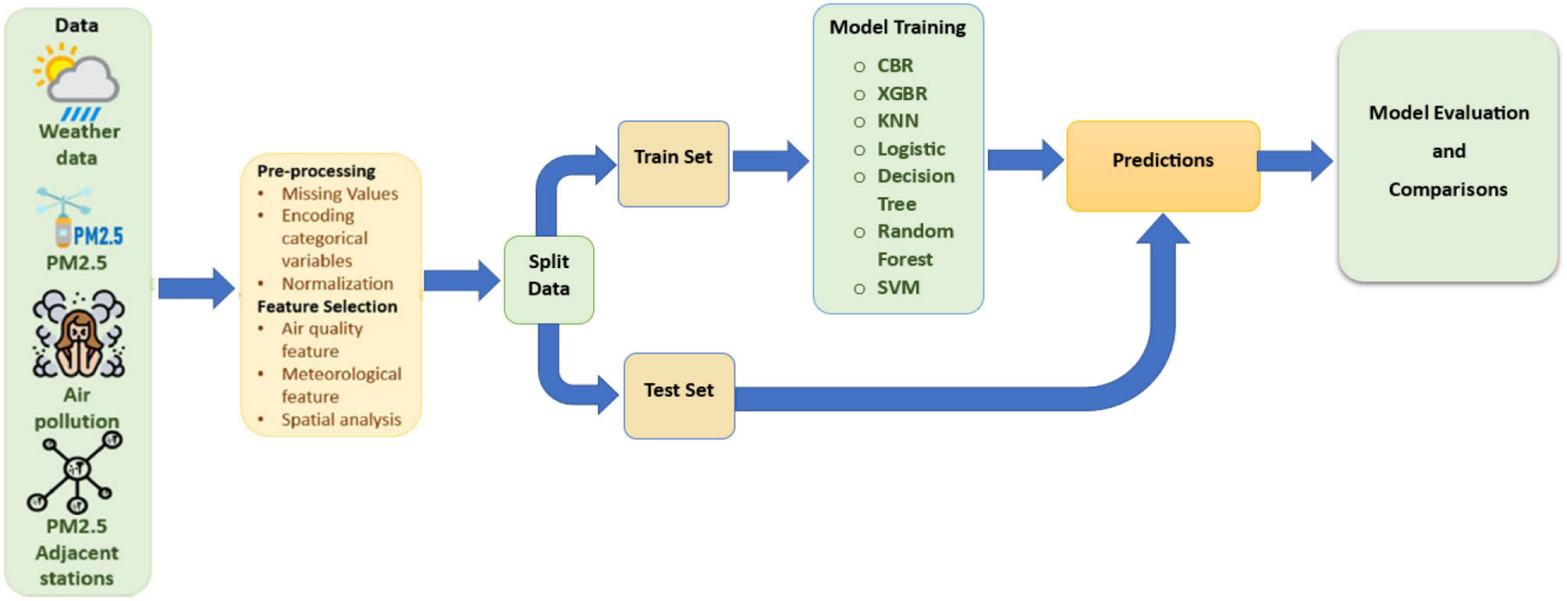
Workflow for system modeling.

The datasets were collected from different sites that need to be converted into a generalized format to recover from missing and null values. Then the ML algorithms are applied to extract patterns and find the highest accuracy. Figure 5 represents the complete workflow of the System modeling.

Cat Boost Regressor and Extreme Gradient Boosting Regressor were used to make PM2.5 predictions then the best was selected to make PM2.5 predictions on the cities not included in the Dataset. Then the Static Variables and Time-series Data for those Cities are added, and the feature engineering is done when training. K-Nearest Neighbor, Logistic Regression, Support Vector Machine, Decision Tree, and Random Forest Classifier are used to make Air Quality Index status predictions, whether Air is 'Good, Moderate, Severe, Unhealthy, Very Unhealthy or Hazardous' based on the Median PM2.5. A system is created where you will need to enter the value of the PM2.5 then the results will be the AQI Status.

### 3.6. Hyperparameter tuning

The K-fold for the Cat Boost Regressor is set to 5 splits, with 1,000 iterations. The loss function is Root Mean Square Error (RMSE), with 100 early stopping rounds and verbose being false for the latest and future predictions. The verbosity of the XGB Regressor is set to zero for the latest forecasts and future projections.

### 3.7. Performance evaluation

The two metrics that are most frequently employed are RMSE (root mean squared error) and MAE (Mean Absolute Error), which are based on the discrepancy between the predicted result and the true value. Performance validation introduces bias when the data set is partitioned, taught, and tested simply once. This suggests that the results acquired from the testing dataset might no longer be valid if the testing subset is changed.

To measure differences between an estimator's anticipated value and the actual value, one uses RMSE (Root Mean Square Error). The term “root mean square error” can also describe this error measurement method. It establishes the importance of the error. A measure of mistakes between paired observations representing the same phenomenon is called MAE (Mean Absolute Error). The ratio of a genuine positive to the total of a false positive and false negative is known as Sensitivity. The ratio of a true negative to the total of a true negative and a false positive is known as Specificity.

## 4. Experiment and results

Evaluation Models used for predicting PM2.5 concentrations for South African Cities.

### 4.1. Cat boost regressor

**Figure 6:**
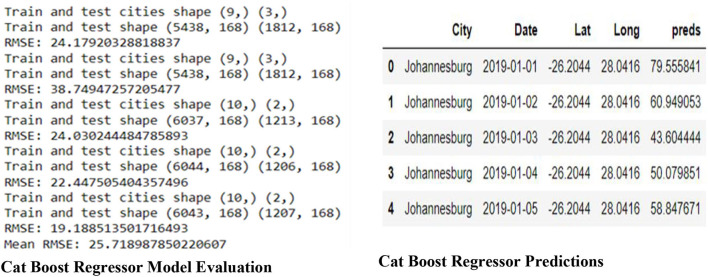
Cat Boost Regressor model evaluation and predictions.

Figure 6 shows the Model Evaluation for the Cat Boost Regressor, which includes the data shape of the train and test data frame, the RMSE (Root Mean Square Error) for each in five steps, and the overall mean RMSE of the five steps. The predictions of the Cat Boost Regressor on the Training data, the predictions are saved under the column named 'preds.

#### 4.1.1. Cat boost actual PM2.5 vs. predicted PM2.5

**Figure 7:**
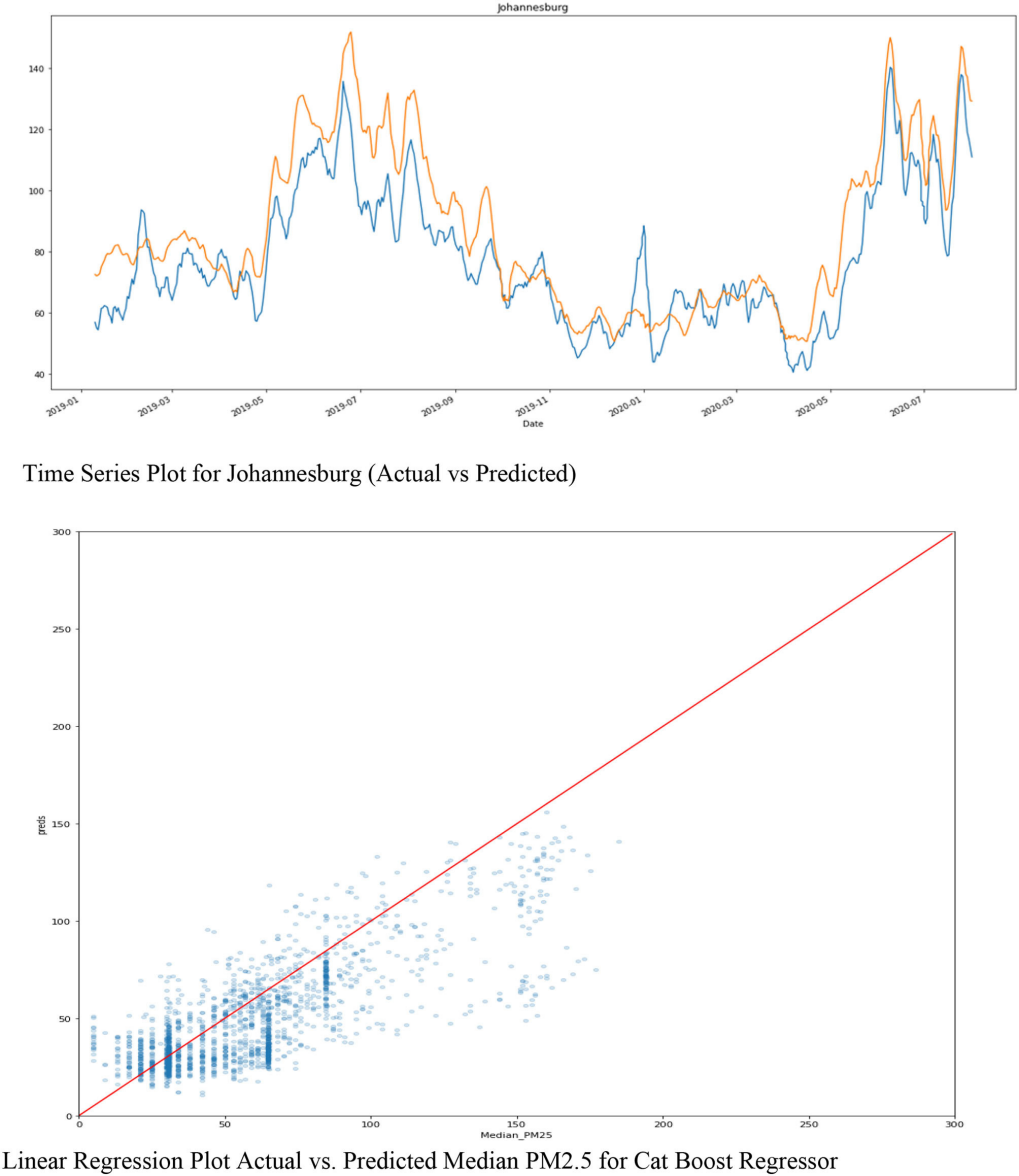
Time series and linear regression plots of Cat Boost Actual vs. Predicted PM2.5.

The time series plot for Johannesburg “SMOOTHED” of the 'Predicted (orange) vs. Actual (blue)' for Johannesburg city stations of PM2.5 is depicted in Figure 7. The linear regression plot shows that the predicted and the actual are not so far apart. They are almost the same; therefore, they have a better correlation.

### 4.2. XGB (extreme gradient boosting) regressor

**Figure 8:**
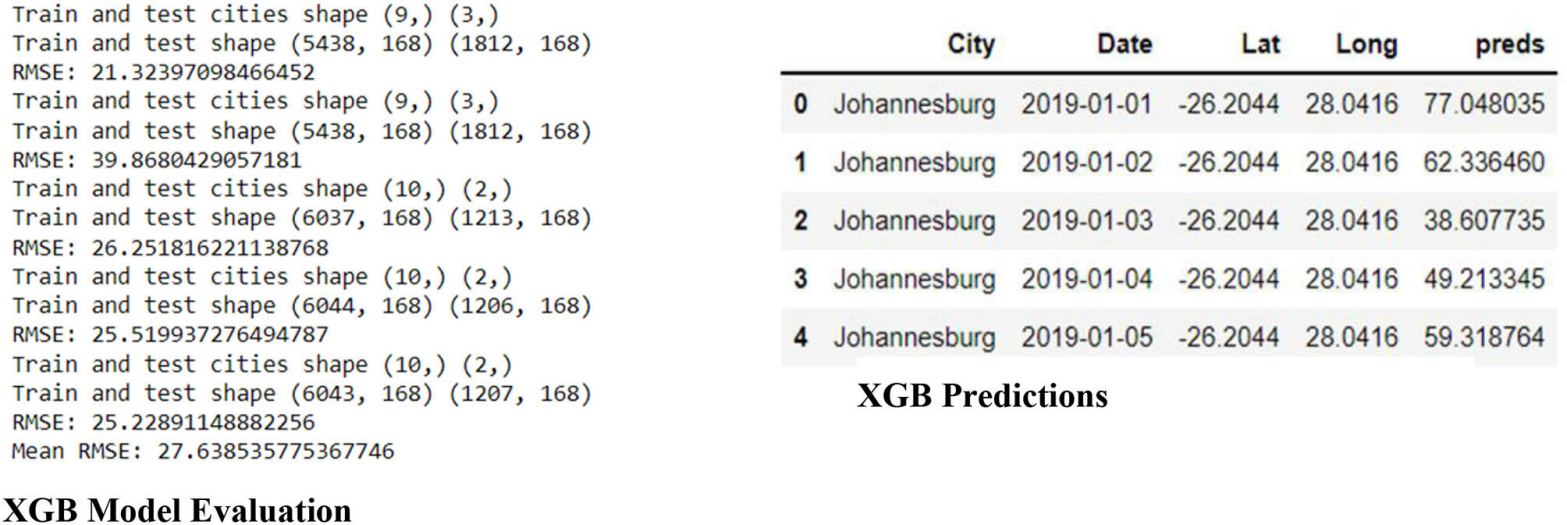
XGB evaluation and predictions.

Figure 8 shows the Model Evaluation for XGB, which includes the data shape of the train and test data frame and the RMSE (Root Mean Square Error) for each in five steps, then the mean RMSE of the five steps. In addition, Figure 8 shows the predictions of the XGB on the Training data. The predictions are saved under the column named “preds”.

#### 4.2.1. XGB actual PM2.5 vs. predicted PM2.5

**Figure 9:**
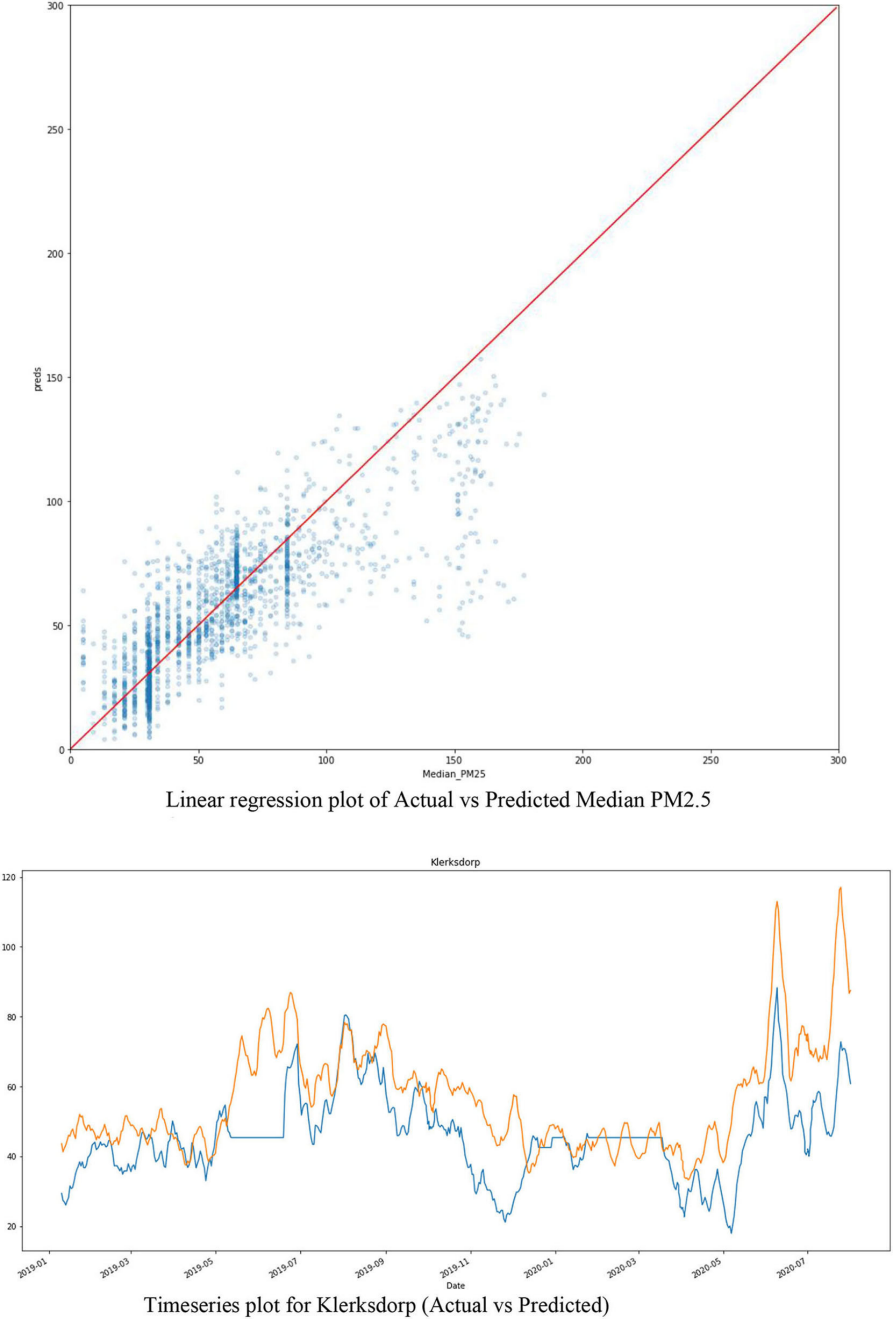
Time series and linear regression and time series plots of XGB actual vs. Predicted PM2.5.

The linear regression plot of Figure 9 shows an excellent correlation between the Actual (Median_PM25) and Predicted (Preds) PM25. Furthermore, Figure 9 shows the smoothed time series plot of the 'Predicted (orange) vs. Actual (blue)' for the Klerksdorp station of PM2.5.

### 4.3. Parameter analysis results

Table 2 shows both regression models used when training and testing the dataset, and the CBR model performed better.

**Table 2:** RMSE of regression models used for predictions.

	**CBR**	**XGBR**
RMSE	25.72	27.64

### 4.4. Predictions on South African cities which were not included in the dataset

**Figure 10:**
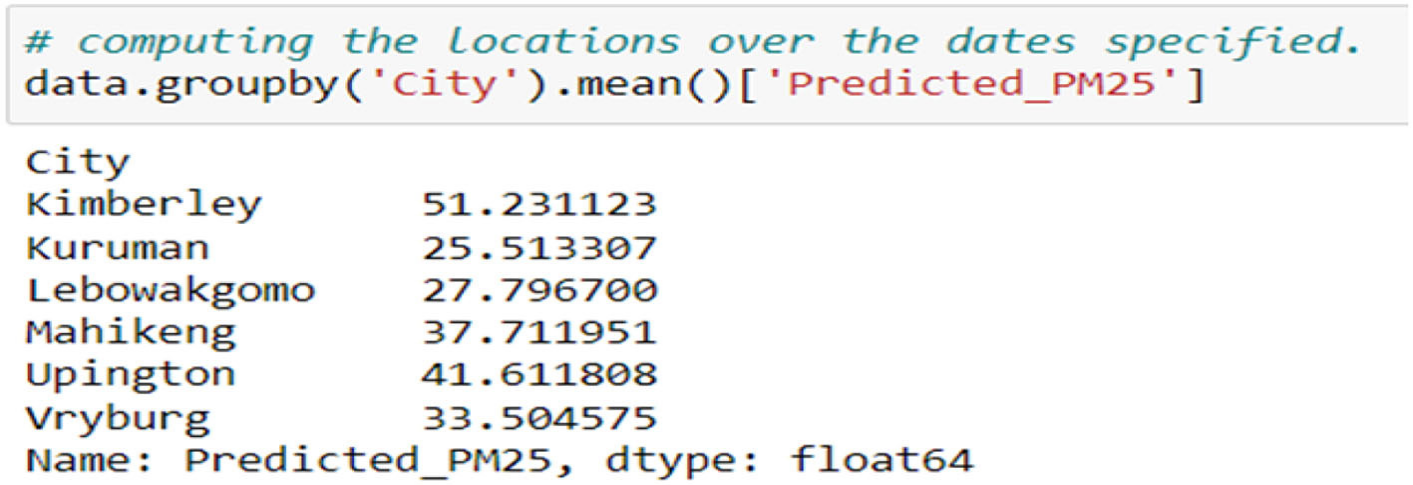
Predictions of cities with no stations.

Figure 10 shows the mean of the predicted PM2.5 concentrations of the cities that do not have the stations. Cat Boost Regressor was used to make PM2.5 predictions because it had better accuracy. These cities had no historical data, and these predictions are made based on the other cities' recordings and based on the neighboring cities. Therefore, it was best to use a better-accuracy model to make these predictions.

### 4.5. Future predictions on South African cities

**Figure 11:**
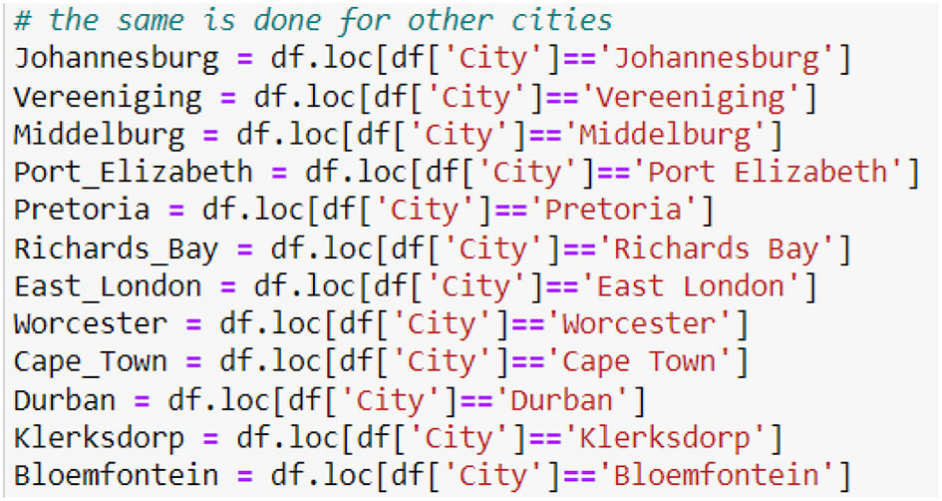
Clustering the city data.

Each City's data is clustered from the data with the PM2.5 concentrations for all the cities to make future predictions (Figure 11).

**Figure 12:**
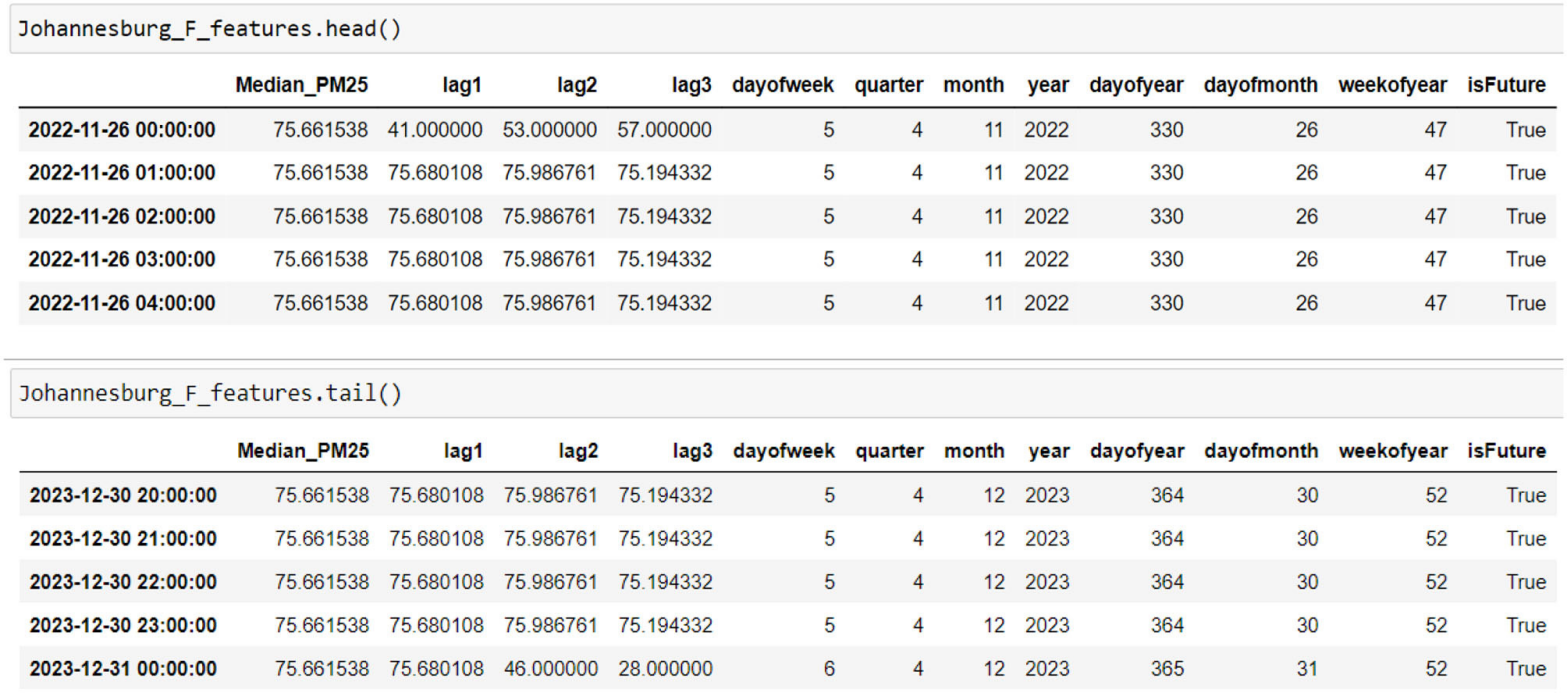
Future PM2.5 predictions (data frame).

Figure 12 shows the head and tail of the data frame Johannesburg_F_features, which contains the predicted PM2.5 concentrations for Johannesburg from 26 November 2022 to 31 December 2023 (Figure 12).

**Figure 13:**
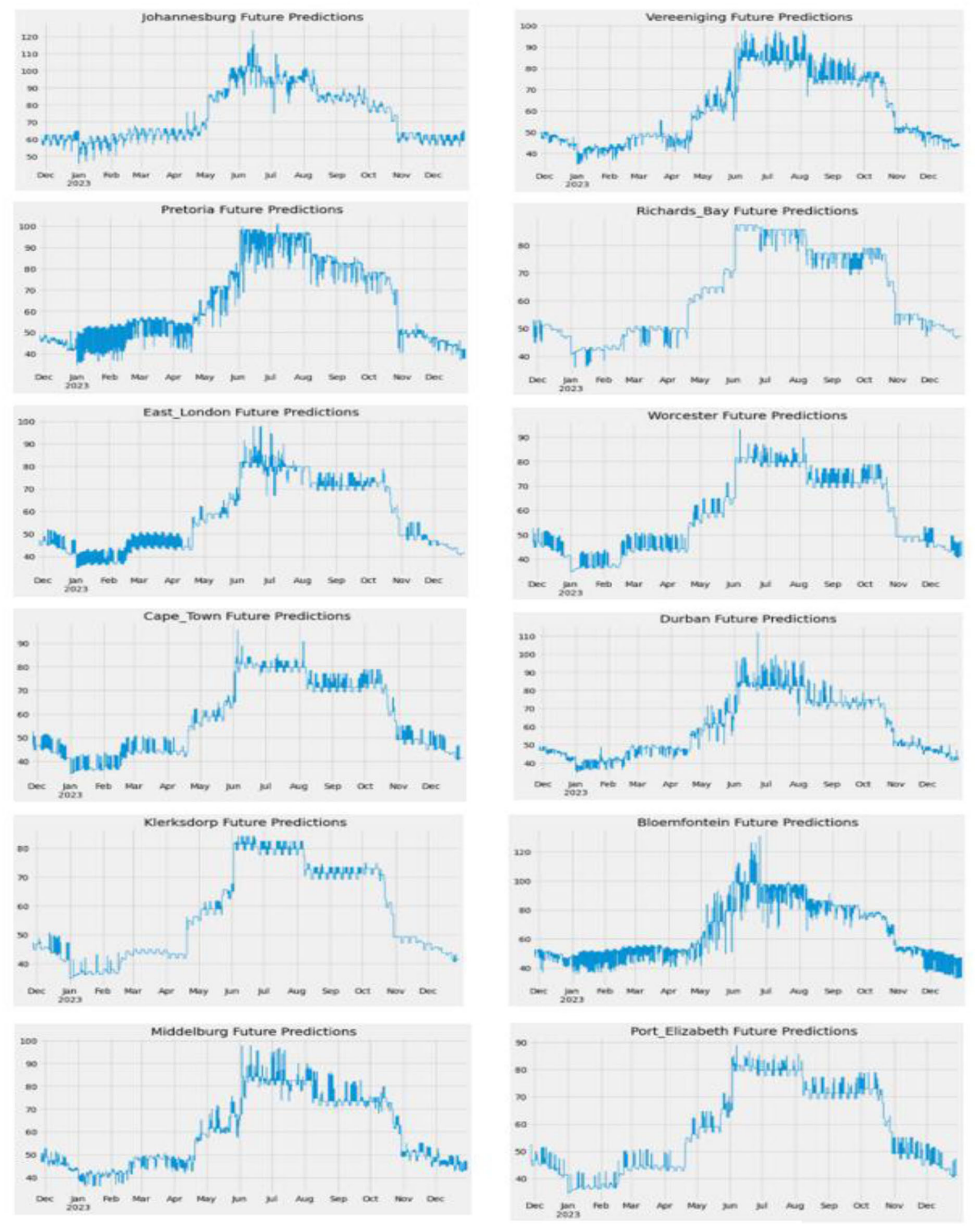
Future PM2.5 predictions of South African cities (Graph).

Figure 13 shows the Future Predictions of PM2.5 concentration from 26 November 2022 to 31 December 2023, using the XGB Model. Any of these two models, Cat Boost and XGB, had the best accuracy, and there was not much of a difference between them. Therefore, both were used to make different predictions.

### 4.6. Evaluating models used for predicting the air quality index status

From Table 3, Decision Tree and Random Forest have 100% accuracy in predicting the AQI Status. More data was needed to check if the data changed, the accuracy would remain the same.

**Table 3:** Results from the models for predicting AQI status.

**Model**	**Accuracy**	**Sensitivity**	**Specificity**	**MAE**	**MSE**	**RMSE**
RF	100.0	1.0	1.0	0.0	0.0	0.0
LR	98.957	1.0	1.0	0.003862	0.003862	0.062144
SVM	98.881	1.0	0.973	0.012040	0.012040	0.109727
KNN	99.986	1.0	1.0	0.000227	0.000227	0.015072
DT	100.0	1.0	1.0	0.0	0.0	0.0

**Figure 14:**
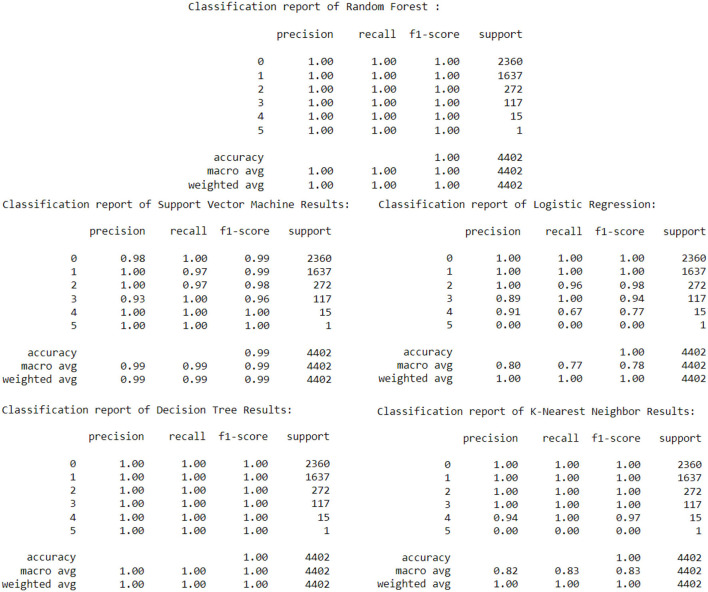
Classification reports of models used.

Figure 14 shows the classification reports of the models used for predicting the AQI Status.

### 4.7. Making prediction results for the AQI status

**Figure 15:**
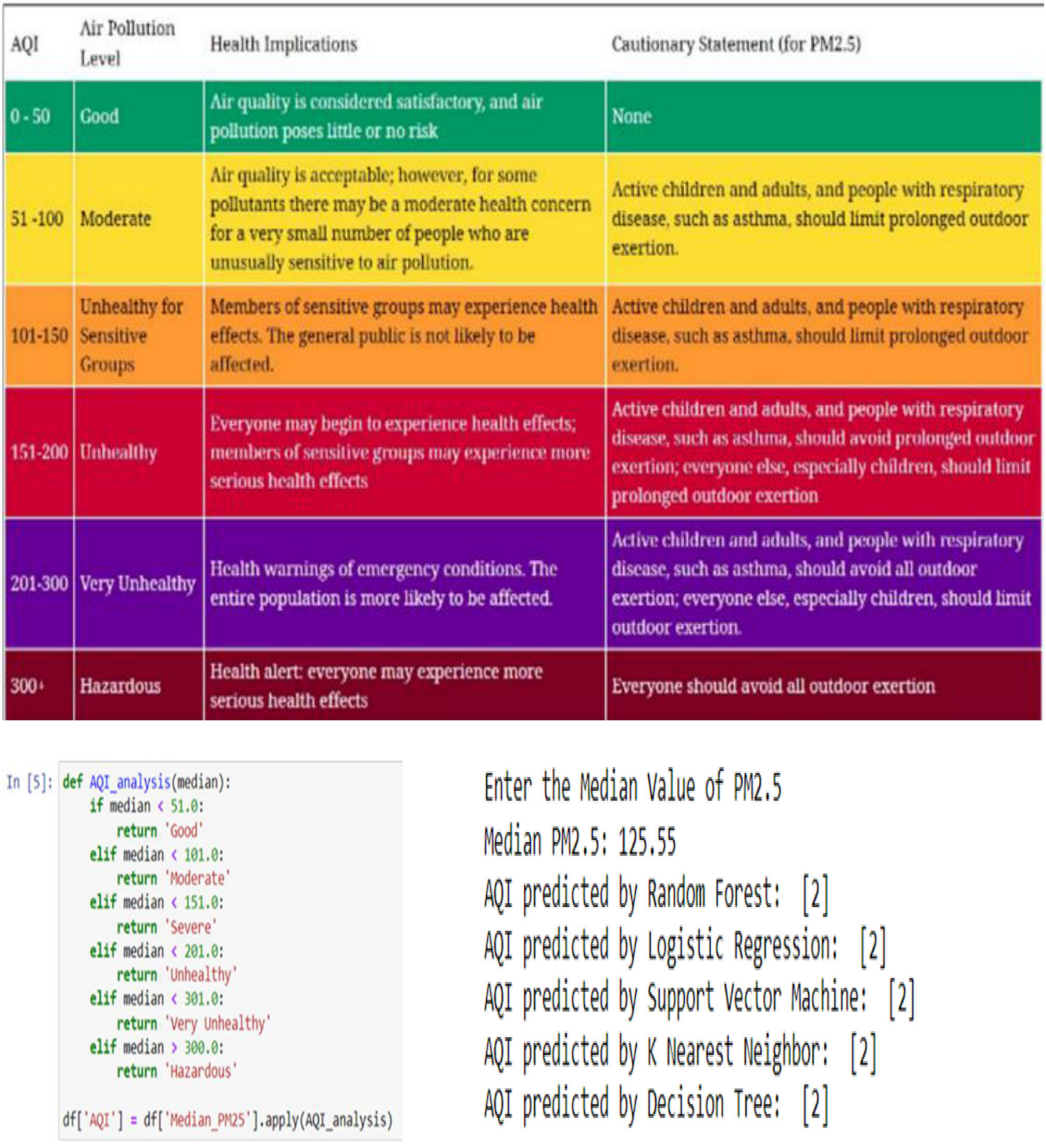
AQI threshold (https://aqicn.org/data-platform/covid19/); AQI analysis function; AQI status predictions.

Figure 15 depicts the AQI threshold, AQI analysis function (defined based on the AQI Threshold), and AQI status predictions respectively. “Good”: 0, “Moderate”: 1, “Severe”: 2, “Unhealthy”: 3, “Very Unhealthy”: 4, “Hazardous”: 5.

Regarding AQI Status Predictions shown in Figure 15, when making predictions for the AQI status, an input value of PM.25 is required to output the prediction. As we can see from Figure 15, the input entered for the PM2.5 median value was 125.55, then each model had their own predicted output, and they all indicated that the forewarned is 2, which means that the air is severe.

## 5. Discussion of results

The likelihood of PM2.5 surpassing the healthy level is predicted using regression models. Two regression models, Cat Boost Regressor and Extreme Gradient Boosting Regressor, were implemented for the PM 2.5 prediction. These models achieved reasonably good Accuracy scores of over 0.6 and were both often correct for over 0.9 of the time. To predict the Air Quality Status, K-Nearest Neighbor, Logistic Regression, Support Vector Machine, Decision Tree, and Random Forest Classifier, five classification models were implemented with an excellent Accuracy Score of over 0.98 to predict when provided the PM2.5 Level.

To assess the high-level relevance of traits, the Mean RMSE of all models used is compared, and the actual is compared to the predicted. The lagged inputs played a significant role in predicting the PM2.5 and the AQI status, as many of them were selected and used by models when predicting. According to the results, Cat Boost Regressor was the best model to predict PM2.5. Furthermore, for AQI status, Random Forest Classifier and Decision were equally the best.

## 6. Comparison of this work with existing research

In this study, SVM, Random Forest, and KNN performed better with accuracy of 98.88%, 100%, and 99.99%, respectively, compared to the same models by Akiladevi et al. (2020), which achieved the accuracy of 70%, 99%, and 97%, respectively. The Decision Tree performed best in both cases, with an accuracy of 100%.

Cross-validation, XGB, the second fold, had the highest RMSE of 39.86 compared to the XGB used by Zamani Joharestani et al. (2019), which achieved 13.58.

Gupta et al. included models with an accuracy of 99.88% for CatBoost regression, 92.40% for SVM, and 91.99% for Decision Tree. In contrast, this study has the accuracy of CatBoost regression, 98.88% for SVM and 100% for Decision Tree.

Generally, the models used in this work perform better on our datasets when compared to existing works using similar models, as shown in Table 4.

**Table 4:** Comparing this work with existing work.

**Models**	**This study**	**Akiladevi et al., 2020**
SVM	98.88%	70%
Random forest	100%	99%
KNN	99.99%	97%
DT	100%	100%
Models	This study	Zamani Joharestani et al., 2019
XGB	39.86	13.58
Models	This study	Gupta et al., 2023
SVM	98.88%	92.40%
Decision Tree	100%	91.99%

## 7. Conclusion

This study focused on predicting the concentration of PM2.5 pollutants in South African cities. The proposed machine learning models are intended to forecast the probability that PM2.5 would surpass the established threshold or not. At various heights above the ground along a vertical axis, meteorological data and air pollutant PM2.5 features are carefully considered. The forecasting ability of the models may be improved by incorporating other characteristics into Google Earth Engine that further extract meaningful information from the data. A higher forecast performance may be possible if more extensive and reliable data are provided. More complex models, like deep learning techniques, may improve prediction accuracy with a larger dataset.

Several models were used, and regression models used included Cat Boost Regressor and Extreme Gradient Boosting Regressor; the performance measure used is an RMSE (Root Mean Square Error). Classification models included K-Nearest Neighbor, Logistic Regression, Support Vector Machine, Decision Tree, and Random Forest Classifier, which were compared using the MSE (Mean Square Error), MAE (Mean Absolute Error), and RMSE (Root Mean Square Error) parameters for predicting the Air Quality Index (AQI) Status. The results show that the proposed hybrid model is more accurate than the solo models, proving its superiority. The suggested method can be used in the future to forecast data from other cities. Using prediction, we may also identify the polluted area and its root cause. Some pollutants pose a severe threat to human health in the future.

## 8. Future work

The data used in this investigation is static. Interestingly, the site offered daily updates to the data. Leveraging real-time data analysis through the cloud to create better results for improved performance shall be considered in the future extension of this work. Moreover, the models used in this work will be evaluated on more datasets from Nitrogen Dioxide (NO2), Ozone (O3), Sulfur Dioxide (SO2), Carbon Monoxide (CO) pollutants. Furthermore, Deep learning methods and Ensembled methods shall be consider for PM2.5, PM10 and other pollutants indicated above.

## 9. Limitation

Not all the South African cities were included in the Dataset. This is because the ones included are the ones that are only having the stations. Even though it was possible to make predictions of the selected cities, the comparison could not be made for all the cities in South Africa since there are no recorded readings for some cities.

## Data availability statement

The original contributions presented in the study are included in the article/supplementary material, further inquiries can be directed to the corresponding author.

## Author contributions

Study conception and design, analysis and interpretation of results, and draft manuscript preparation: IO and TM. Data collection: TM. All authors reviewed the results and approved the final version of the manuscript.
